# A Proof-of-Concept Study: Simple and Effective Detection of P and T Waves in Arrhythmic ECG Signals

**DOI:** 10.3390/bioengineering3040026

**Published:** 2016-10-17

**Authors:** Mohamed Elgendi, Marianna Meo, Derek Abbott

**Affiliations:** 1 Department of Obstetrics & Gynecology, University of British Columbia and BC Children’s & Women’s Hospital, Vancouver, BC V6H 3N1, Canada; 2 Electrophysiology and Heart Modeling Institute, (IHU LIRYC), Bordeaux 33604, France; marianna_meo@libero.it; 3 School of Electrical and Electronics Engineering, University of Adelaide, Adelaide SA 5005, Australia; derek.abbott@adelaide.edu.au

**Keywords:** mobile health, affordable healthcare, numerically-efficient algorithms

## Abstract

A robust and numerically-efficient method based on two moving average filters, followed by a dynamic event-related threshold, has been developed to detect P and T waves in electrocardiogram (ECG) signals as a proof-of-concept. Detection of P and T waves is affected by the quality and abnormalities in ECG recordings; the proposed method can detect P and T waves simultaneously through a unique algorithm despite these challenges. The algorithm was tested on arrhythmic ECG signals extracted from the MIT-BIH arrhythmia database with 21,702 beats. These signals typically suffer from: (1) non-stationary effects; (2) low signal-to-noise ratio; (3) premature atrial complexes; (4) premature ventricular complexes; (5) left bundle branch blocks; and (6) right bundle branch blocks. Interestingly, our algorithm obtained a sensitivity of 98.05% and a positive predictivity of 97.11% for P waves, and a sensitivity of 99.86% and a positive predictivity of 99.65% for T waves. These results, combined with the simplicity of the method, demonstrate that an efficient and simple algorithm can suit portable, wearable, and battery-operated ECG devices.

## 1. Introduction

According to the World Health Organization (WHO), cardiovascular diseases (CVDs) are the leading cause of death globally [1]. In recent years, a variety of programs and policies have been implemented in increasingly diverse communities to provide tools, strategies, and other best practices to reduce the incidence of initial and recurrent cardiovascular events [2]. To achieve this goal, the electrocardiogram (ECG) has become the most commonly used biosignal for the prompt detection of CVDs. Additionally, ECGs are used in the initiation of therapy in patients with acute coronary syndromes and the diagnosis of intraventricular conduction disturbances and arrhythmias [3]. However, ECG recordings may be collected with different durations (e.g., over 10 min during one session, for up to 7 days), thus requiring a robust and numerically-efficient algorithm to analyze the long-recorded data and detect their dynamic and characteristic waves [4].

Analysis of the P and T waves of the ECG is essential, as their shape and duration can be severely altered by certain pathologies. Changes in P waves may indicate defective intra-atrial conduction, hypertrophic conditions of the atria and atrio-ventricular conduction, among other abnormalities [5]. In some pathological conditions, T wave morphology may also vary because of altered ventricular activation [5].

Researchers have attempted to employ a variety of methods to detect P and T waves, each with its own strengths and limitations. For example, in [6], the discrete cosine transform (DCT) was used for discrimination of P waves on a few ECG segments from the MIT-BIH database. However, detection generally failed when the amplitude was very small. In [7], a Bayesian sampling algorithm (Partially Collapsed Gibbs Sampler, PCGS) was used to detect P and T waves in the PhysioNet QT interval database. Even though both waves were accurately detected at the same time, their characterization strongly depended on an a priori model assumption that was not verified, especially in pathological conditions. Moreover, it is has a high computational overhead.

In [8], a template-based correlation method was tested on the Physionet QT database. Despite rather satisfactory detection rates, the authors stated that estimation uncertainty is higher when signal quality is poor, thus demonstrating the lack of robustness of the method for noisy signals. The study in [9] proposed a Field Programmable Gate Array (FPGA) based system combined with the identification of the slopes of the waves of interest. The method was validated on the Physikalisch-Technische Bundesanstalt (PTB) diagnostic ECG database from Physionet. Nevertheless, only a portion of the database was examined, and half of the recordings (out of 12 s) was employed for algorithm training.

Li et al. [10] proposed a method for detecting monophasic P and T waves based on quadratic spline wavelets with compact support, but without validation on an ECG database. De Azevedo et al. [11] used a neural network with asymmetric basis functions to extract the features of the P waves on the MIT-BIH database. However, they did not mention the detection rate.

Strumillo [12] demonstrated a nonlinear signal decomposition method based on nested median filters. It was tested on the QT database for detecting T wave offset, but the detection rate was not reported. Martinez et al. [13] presented a generalized method for the discrimination of P and T waves based on quadratic spline wavelets and the derivative of a Gaussian as a smoothing function and tested it on the QT database. The biorthogonal wavelet transform was performed in [14] for P wave detection, but database source is not detailed. Chouhan et al. [15] used the first derivative with adaptive thresholds for simultaneous P and T wave detection.

Most of the algorithms in the literature detect either P or T waves separately. Many attempts have been made to find a satisfying universal solution for P and T wave detection. Moreover, most of these algorithms reported high P and T wave detection performance with high rates after excluding some segments or beats from the used records. Difficulties arise mainly because of the diversity of the P and T waveforms, low signal-to-noise ratio (SNR) and the artifacts accompanying the ECG signals [16], especially in the presence of arrhythmia [17].

The performance of the existing P and T wave detection algorithms is still inefficient and needs to be tested on long recordings rather than short ECG segments, such as those signals found in the well-known MIT-BIH database [18,19]. The recordings included in this dataset present a wide variety of cardiac disturbances, such as such as premature atrial complexes (PAC), premature ventricular complexes (PVC), left bundle branch blocks (LBBB), and right bundle branch blocks (RBBB).

Through this proof-of-concept study, we demonstrate a numerically-efficient and robust algorithm. We show that the proposed algorithm can detect P and T waves, despite the effect of pathological conditions on their properties—in particular, morphology and duration.

## 2. Data

Several standard ECG databases are available for the evaluation of QRS detection algorithms for ECG signals. Most of these databases contain annotated files for R peaks but not for P and T waves. We annotated the P and T peaks in 10 ECG recordings from the MIT-BIH Arrhythmia Database [18,19] to be used in evaluation for the following reasons:

- The MIT-BIH database contains 30-min recordings for each patient, which is considerably longer than the recordings in many other databases, such as the Common Standards for Electrocardiography (CSE) database that contains 10-s recordings [20].
- Arrhythmic ECG signals provided by the *MIT-BIH Arrhythmia Database* are impacted by multiple factors that affect signal quality. For example, we noted premature atrial complexes, non-stationary effects, premature ventricular complexes, low signal-to-noise ratio, left bundle blocks, and right bundle blocks. These challenges provide an opportunity to test the robustness of the P and T wave detection algorithm. These issues are expected to present significant difficulties for any ECG signal analysis algorithm [21].

The selected ten ECG recordings from the MIT-BIH Arrhythmia Database contain a total of 21,702 beats and each was a 30-minute recording. The sampling frequency was 360 Hz with an 11-bit rate resolution over a 10 mV range. The quality of the Leads varied and therefore we selected the Lead with the higher quality signals (Lead I). Annotations of P and T waves in MIT-BIH is described in [22] and can be downloaded from http://www.elgendi.net/databases.htm.

## 3. Methodology

**Figure 1:**
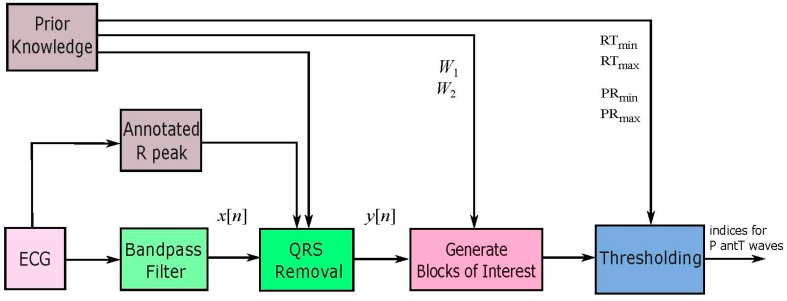
Structure of the proposed P and T waves detection algorithm.

A new numerically-efficient and robust algorithm adapted from the two event-related moving-average filter, discussed in [22], is proposed to detect P and T waves in ECG signals. The structure of the algorithm consists of three main parts as shown in Figure 1. Prior information about the duration of the P and T waves plays a major role in the decision making of the proposed algorithm in both stages: generating blocks of interest and thresholding.

### 3.1. Prior Information Analysis

The P and T wave detector is expected to improve the overall the performance and detection accuracy based on the prior duration information. In a normal (sinus) heart rhythm, each heartbeat consists of five distinct waves (P, Q, R, S, T). These distinct waves have a very specific sequential order and duration. Any deviation from the normal range for sinus rhythm is considered arrhythmic. Clifford et al. [17] provided information about the normal limits for the main events within the EGG, for a healthy male adult at 60 beats per minute (bpm), shown in Table 1.

Our algorithm depends on the estimate of the event duration before processing the ECG signal. For example, the expected durations of the main events of the ECG signals serve to assist the feature extraction phase. Moreover, decision making of the algorithm is supported by these durations. To illustrate this in more detail, by knowing that the P wave duration in a normal healthy subject varies from 33 to 47 samples (sampling frequency of 360 Hz), the values of *W*_1_ will be set based on this prior information. Setting the value of *W*_2_ depends on the expected duration of one heartbeat. In healthy subjects, this translates into approximately 1 s, therefore resulting in 360 samples in length (sampling frequency of 360 Hz). It is known that heartbeats vary from person to person and this is a rough estimate.

The QT, PR and RT intervals are dependent on the heart rate in an obvious way, the faster the heart rate the shorter the QT, PR and RT intervals; the consideration of this knowledge improves the detection of P and T waves. According to Table 1, the average duration of P wave is 110 ms; however, the minimum PR interval is 120 ms for a healthy subject (60 bpm). Certainly, in arrhythmic ECG signals the PR wave will reduce. Therefore, the minimum PR interval (*P*_min_*R**_i_***_+ 1_) will be considered as (110 ms) × *R_i_R_i_*_+ 1_. Additionally, the *P*_max_*R_i_*_+ 1_ equals (560 ms) × *R_i_R_i_*_+ 1_. The value of the 560 ms was obtained from Table 1, for a total duration of PQ and QT_c_ intervals.

### 3.2. Bandpass Filter

Morphologies of normal and abnormal QRS complexes differ widely. The ECG signal is often corrupted with noise from many sources: 50/60 Hz from power-line interference, EMGs from muscles, and motion artifacts, which have been discussed in [23]. Therefore, band pass filtering is an essential first step for nearly all QRS detection algorithms. The purpose of band-pass filtering is to remove the baseline wander and high frequencies that do not contribute to P and T wave detection.

**Figure 2:**
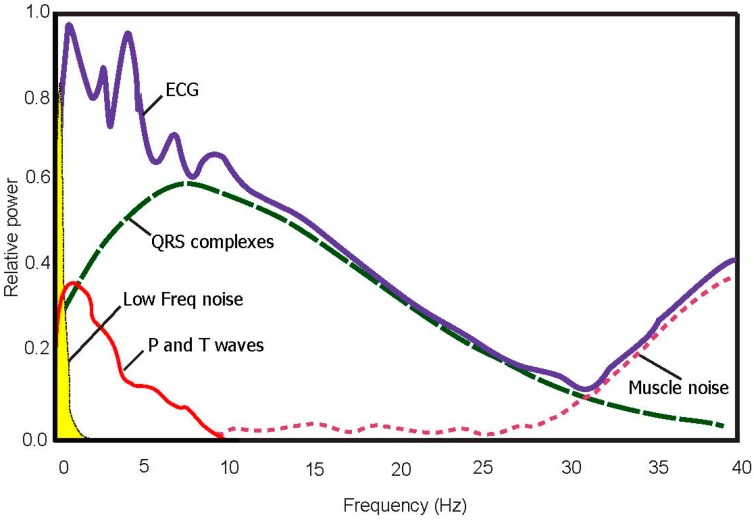
**Power spectra of noisy ECG signal (100 beats).** The optimal frequency band to detect T waves is 0.5–10 Hz.

A bidirectional Butterworth (i.e., “Butterworth filters”) bandpass filter is used based on the recommendation in [24]. The filter offers the required transition-band characteristics at low coefficient orders, which facilitates efficient implementation [24]. The main frequencies of the P and the T wave lie in the range of 0.5 Hz to 10 Hz as shown in the power spectra shown in Figure 2 and confirmed in [25]. Therefore, in this study, the baseline wander and high frequencies, which does not contribute to P and T wave detection, is removed using a second-order Butterworth filter with passband 0.5–10 Hz.

### 3.3. QRS Removal

The first stage of QRS removal is to detect R peaks, and then the QRS complex can be removed from each beat to make the P and T wave dominant. The removal is carried out by setting the values before and after the R peak to zeros. Note that annotation of the R peaks has already been completed and provided in the *MIT-BIH Arrhythmia Database,* and thus there is no need to detect R peaks.

**Figure 3:**
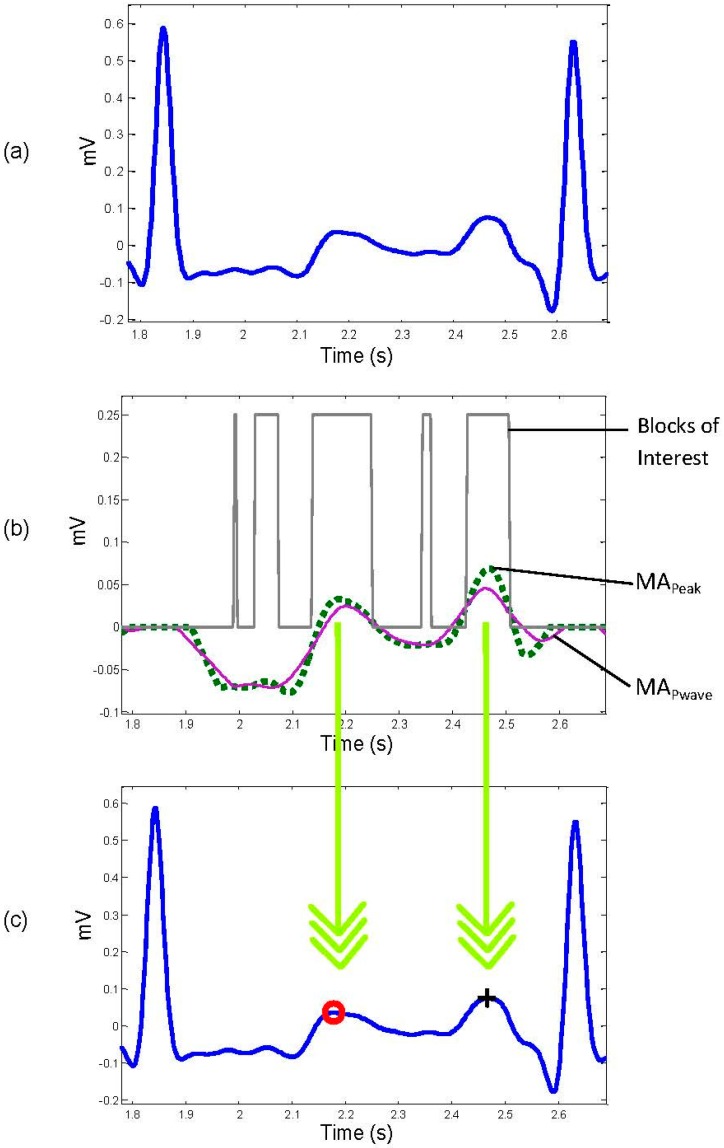
**Application of two moving averages to detect P and T waves.** (**a**) Filtered RR ECG signal with Butterworth bandpass filter; (**b**) generating blocks of interest after using two moving averages: the dotted line is the first moving average and the solid line is the second moving average; (**c**) the detected P and T waves after applying the thresholds. The red “o” identifies the T wave, while the black “+” symbol identifies the P wave.

The signal is set to zero in a range equal to 0.083 ms before the R peak and for 0.166 ms after the R peak. Figure 3b shows the result of removing QRS complexes from the filtered signal of Figure 3a.

### 3.4. Select Potential Blocks

The moving averages will demarcate the onset and offset of the potential P and T waves based on a prior knowledge of P and T wave durations. Healthy adults have a normal duration for P waves that varies within the range of 110 ± 20 ms, at a heart rate of 60 beats per min [17], while the normal duration for the QT interval varies within the range of 400 ± 40 ms.

Suitable window sizes of the moving averages are set by the average duration of each event. For example, the window size of the P wave duration is approximately 110 ms, and the average window size corresponding to the QT_c_ duration is approximately 400 ms. The window size of the two moving averages are set based on the P wave duration; it is expected that the P wave duration is smaller than the T wave duration. Therefore, when the two moving averages (Equations (1) and (2)) capture the P wave, it also captures the T wave. To explain further, the second moving average with a larger window size is used as a threshold for the first moving average, which captures the P and T waves simultaneously.

**(i)** **First moving average**: The first moving-average integration is used to demarcate the P and T waves with a sharp wave, shown as the dotted line in Figure 3b.

(1) $${\text{MA}}_{\text{Peak}}[n]=\frac{1}{W_{1}}(x[n-(W_{1}-1)/2]+....+x[n]....+x[n+(W_{1}-1)/2])$$

where *W*_1_ = 55 ms is half the window width of the P wave. In order to demarcate the small P and T duration for severe cases of arrhythmia, a smaller window size is chosen, rather than the expected window size set for healthy subjects. 

**(ii) Second moving average:** The purpose of the second moving average (MA_pwave_) is to be used as a threshold for the first moving average MA_peak_ integration, shown as the solid line in Figure 3b:

(2) $${\text{MA}}_{\text{Pwave}}[n]=\frac{1}{W_{2}}(x[n-(W_{2}-1)/2]+....+x[n]....+x[n+(W_{2}-1)/2])$$

where *W*_2_ = 110 ms is the window width of the P interval.

As discussed above, the window size of the first moving average should be less than the average healthy duration for the P wave—which is half of the P wave duration—while the window size of the second moving average equals the average healthy P wave duration. The first moving average will demarcate the P and T waves—especially in cases of arrhythmia with smaller durations. The second moving average then works as a threshold for the first moving average.

When the amplitude of the first moving-average filter (MA_peak_) is greater than the amplitude of the second moving-average filter (MA_pwave_), that part of the signal is selected as a block of interest, as follows: **IF** MA_peak_[*n*] > MA_pwave_ **THEN** Blocks[*n*] = 0.25 **ELSE** Blocks[*n*] = 0 **Endif**. Figure 3b shows the result of applying the two moving averages.

**Figure 4:**
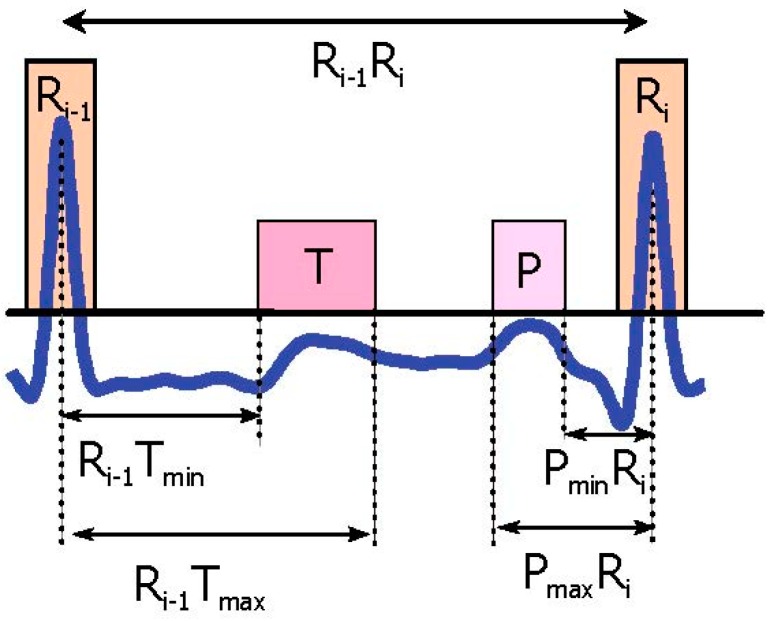
**P and T wave time occurrence with respect to the current R peak and next the R peak**. The *R_i_*_− 1_*T*_min_ represents the minimum interval between the T wave and current the R peak, while *R_i_*_− 1_*T*_max_ is the maximum interval between the T wave and the current the R peak. Here, *P*_min_*R_i_* represents the minimum interval between the P wave and next the R peak, and *P*_max_*R_i_* stands for the maximum interval between the P wave and next the R peak.

**Figure 5:**
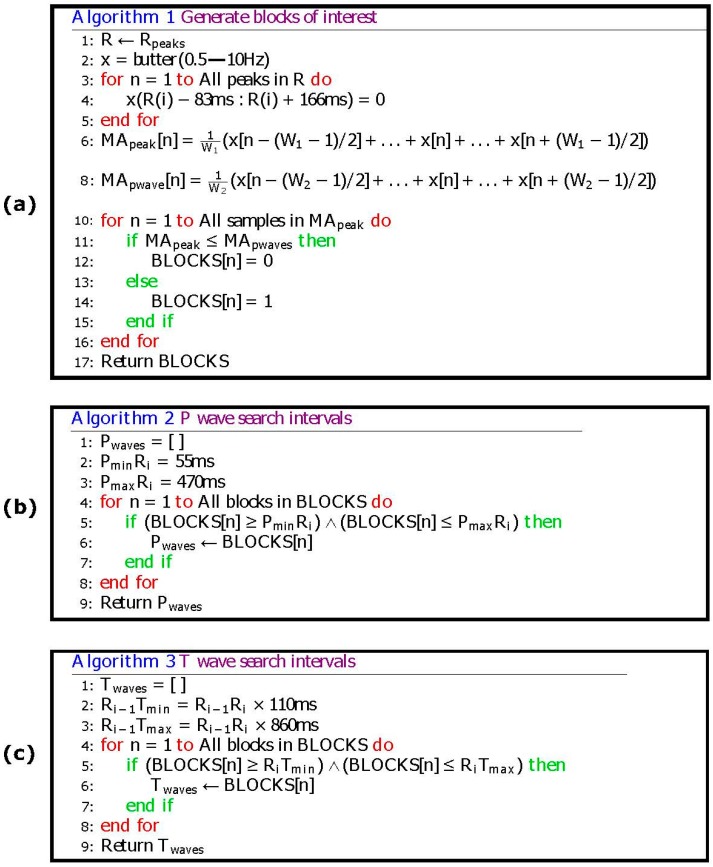
Pseudocode of the proposed P and T waves detection algorithm. The *R_i_*_− 1_*T*_min_ represents the minimum interval between the T wave and current the R peak, while *R_i_*_− 1_*T*_max_ is the maximum interval between the T wave and the current the R peak. Here, *P*_min_*R_i_* represents the minimum interval between the P wave and next the R peak, and *P*_max_*R_i_* stands for the maximum interval between the P wave and next the R peak. (**a**) Generating blocks of interest (**b**) P wave search intervals (**c**) T wave search intervals.

One RR interval shown in Figure 3b demonstrates the idea of using two moving averages to generate blocks of interest. Only blocks with relative positions from P and T waves to the R peaks are considered, an thus, as shown in Figure 4 not all generated blocks of interest are potential P and T waves. In Figure 5a the pseudocode for generating blocks of interest can be seen.

**(iii) Reject noisy blocks:** The blocks associated with small width are considered as blocks caused by noise. Blocks that are smaller than half of the expected size for P waves are rejected. Because the T waves are wider than the P waves, potential T waves are still present. The expected size of the P wave is based on the statistics for healthy adults, as described in [17] which varies from 90 ms to 130 ms. Blocks that are smaller than half of the width of *W*_1_ are rejected for the P wave.

Based on the duration of P and T waves provided in Table 1, the ratio of the P wave duration to T wave duration is 3:5, thus the 0.75 and 1.25 values were used as percentages of the *W*_1_. This corresponds to:

(3) $$P_{Block}=0.75\times W_{1}$$

Similarly, T waves in arrhythmia ECG signals are smaller than in healthy people. Therefore, the block size of T wave will be larger than the block size of P wave. This corresponds to:

(4) $$T_{Block}=1.25\times W_{1}$$

If *P*_Block_ and *T*_Block_ have been set equal to *W*_1_, the results will be close to the reported ones. The fact the P wave duration is smaller than T wave duration supports the idea of decreasing the expected P wave compared to the T wave. Note that the *P*_Block_ will also capture the small T wave durations, as demonstrated in Equations (3) and (4).

### 3.5. Thresholding

In this step, we have a number of blocks between RR interval that are ready to be considered as P and T waves. A threshold, based on the Euclidean distance between the R peaks and anticipated blocks of P and T waves, is applied to filter these blocks and pick only the blocks that contain P and T waves. Block detection occurs in three possible scenarios:

- No blocks detected: No detection of P or T wave in the processed RR interval.
- One block detected: Most likely the P and T waves are merged within one block.
- More than one block detected: Most likely the signal is noisy and therefore multiple blocks are generated. This step has two sub steps:
  ❖ *P wave detection*. If the distance between the maximum value of the block, and the nearest R peak is within a predefined range (which is based on prior knowledge of the PR interval), then the maximum value of the block is the P wave as shown in Figure 4. (Figure 5b demonstrates the pseudocode).
  ❖ *T wave detection*. If the distance between the R peak and the maximum value of the next block is within a predefine range (which is based on prior knowledge of the RT interval), then the maximum value of the next block is the T wave as shown in Figure 4. (Figure 5c demonstrates the pseudocode).

In some cases, there is more than one block within the acceptable range for a P or a T wave. In these cases the block that contains the wave with the maximum amplitude is selected. The two down arrows between Figure 3b,c represent the projection of the maximum amplitudes within the considered blocks on the original ECG signal.

## 4. Results

**Figure 6:**
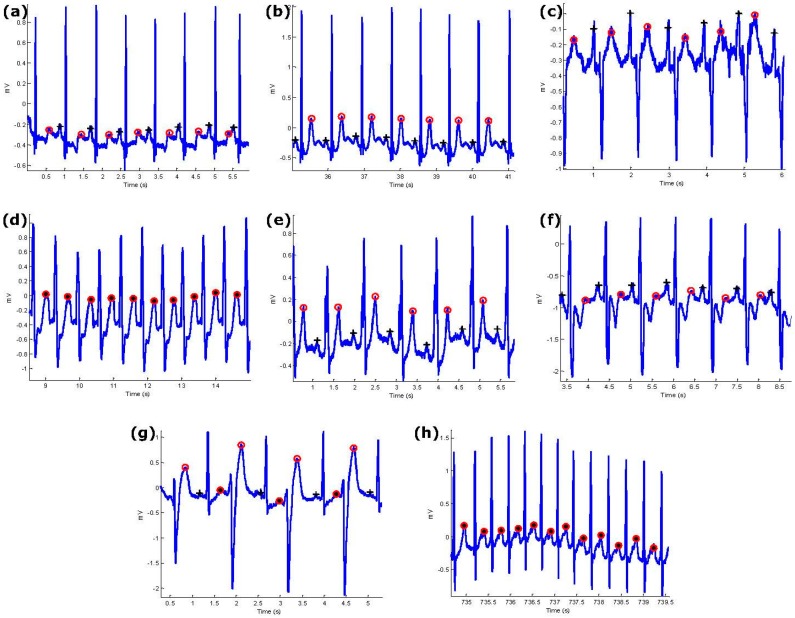
**Performance of the proposed P and T detection algorithm**. The algorithm succeeds in detecting P and T waves in ECG signals that contain (**a**) normal sinus rhythm without U waves; (**b**) normal sinus rhythm with U waves; (**c**) normal sinus rhythm with negative polarization; (**d**) LBBB beats with merged P and T waves; (**e**) LBBB beats; (**f**) RBBB beats from record 118; (**g**) PVC beats from record 200; (**h**) PAC beats from record 209. Here, “+” represents the P wave and “o” represents the T wave while the asterisk represents merged P and T waves.

P and T waves are successfully detected using the proposed algorithm, including merged P and T waves, LBBB, RBBB, PVC, and PAC, in arrhythmic ECG signals from the *MIT-BIH* *Database.* Moreover, the algorithm combated different types of noise such as high-frequency, noise baseline wander, and low SNR. It can be seen in Figure 6 that the ECG signals contained these challenges. Given these results, the algorithm is promising in detecting P and T waves in noisy ECG signals.

The algorithm is applied across three different types of normal rhythms as seen in Figure 6a–c: (1) without U waves (record 100); (2) with U waves (record 103); and (3) with negative polarization (record 108). The P and T wave in a normal sinus rhythm are relatively easier to detect as the P-wave, QRS, T wave do exist and are relatively salient to detect [5].

Note that LBBB is the result of conduction delays or blocks at any site in the intraventricular conduction system, including the main LBBB and the *bundle of His*. The consequence of an LBBB is an extensive reorganization of the activation pattern of the left ventricles [5]. The proposed algorithm successfully detected normal and merged P and T waves in two types of LBBBs: (1) LBBB beats with merged P and T waves (record 109), as shown in Figure 6d, and (2) LBBB beats with normal P and T waves (record 111), as shown in Figure 6e.

However, RBBB is the result of conduction delay in a portion of the right-sided intra-ventricular conduction system. The delay can occur in the main RBBB itself, in the bundle of His, or in the distal right ventricular conduction system. RBBBs may be caused by a minor trauma such as right ventricular catheterization [5]. As shown in Figure 6f, the proposed algorithm succeeded in detecting the P and T waves in ECG signals of RBBB (record 118).

The PVC is defined as the premature occurrence of a QRS complex which has an abnormal morphology and is followed by a longer RR duration when compared to a normal beat, exceeding 120 ms. A special case of PVC is shown in Figure 6g, which is known as bigeminy, and it takes place when the PVCs occur after every normal beat in an alternating pattern. Clearly shown in the figure, the P and T waves are accurately detected over record 200.

PACs are similar to PVCs; however, they are associated with an irritable focus in the atria, giving rise to a distorted P wave, followed by a superimposed T wave [5]. The proposed algorithm detected the merged P and T waves in PACs (record 209), as shown in Figure 6h.

Overall, the detection rate was satisfactory, specifically when dealing with various arrhythmias and different types of noise. Results are shown in more detail in Table 2 and Table 3 for the 10 annotated ECG signals.

Two statistical parameters were used to evaluate the P and T wave detection algorithm: Sensitivity (SE) and Positive Predictivity (+P), calculated as follows: ${\text{SE}}_{P/T}=\frac{{\text{TP}}_{P/T}}{{\text{TP}}_{P/T}\;+{\text{ FN}}_{P/T}}$ and $+\;P_{P/T}=\frac{{\text{TP}}_{P/T}}{{\text{TP}}_{P/T}\;+{\text{ FP}}_{P/T}}$ where True positive (TP_P/T_): P/T wave has been classified as P/T wave, False negative (FN_P/T_): P/T wave has not been classified as P/T wave, and False positive (FP_P/T_): non-P/T wave has been classified as P/T wave.

Here, SE_P/T_ is defined as the percentage of true P/T waves, which are correctly detected, while +P_P/T_ is defined as the percentage of actual P/T waves. The detection algorithm for the P and T waves is impacted by the quality of the recording and any existing abnormalities (see Table 2 for P and T waves detection results). It can be seen in Table 2 that record 108 and 109 are poor quality signals, which caused a large number of FNs, impacting the algorithm detection rate. Note that FPs were result of the presence of arrhythmia and low signal-to-noise ratios, when compared against FNs in P wave detection. On occasion, PVCs and PACs caused false positives. The ECG recording 108 contained the largest number of false positives. In summary, the overall SE for P waves was 98.05%, and the +P was 97.11%.

The T wave detection results are shown in Table 3 for the same 10 records; the overall SE was 99.86% and +P was 99.65%. As with the P waves, the number of FNs was smaller than the number of FPs and were mostly caused by noise. Note that PVCs often caused the FPs for T waves (see record 108) and LBBBs (see record 109).

## 5. Discussion

The performance of the P and T wave detection algorithm on the *MIT-BIH Database* is shown recording by recording in Table 2. Comparisons to other published detectors are provided in Table 3. Two statistical parameters (SE and +P) were used to evaluate the performance of the proposed P and T wave algorithm. Even though the MIT-BIH Arrhythmia Database includes 48 ECG recordings, most of the P and T detection algorithms published by other researchers used few recordings or segments of these signals, as shown in Table 3. Literature citing P and T wave detection in the *MIT-BIH Database* have limitations, such as the ability only detect certain beats or segments. Perhaps the rationale behind this was that no annotation of P and T waves and there was thus no benchmark.

Table 3 shows that most of the algorithms published in the literature were not applied to full recordings. However, the proposed algorithm was able to successfully handle full recordings with high performance, when compared to recent and well-known publications in the field of study. Essentially, the proposed detection algorithm can combat non-stationary effects, low SNR, PACs, PVCs, LBBBs, and RBBBs, and is thus numerically efficient and has the ability to simultaneously detect P and T waves.

The preliminary results are promising, especially after testing the algorithm on 10 recordings drawn from the *MIT-BIH Database*; however, applying the algorithm on the entire dataset is the next logical step to further test the robustness of the detector. Other arrhythmias such as atrial fibrillation, junctional tachycardia, paroxysmal supraventricular tachycardia atrial flutter, and multifocal atrial tachycardia are the next step in investigating the application of our algorithm. At present, our method represents a simple yet efficient P and T wave detection algorithm that may at the very least improve current ECG-based fitness tracking applications.

Our method follows the Eventogram's building blocks [33] and the TERMA framework [34] for P and T wave detection and can be adjusted to analyze ECG for a particular arrhythmia type by changing the filter type, filter order, moving average type based on the application. Moreover, before performing our analysis, the Chauvenet criterion can be applied [35] to discard outliers or noisy ECG samples. The proposed algorithm is dependent on the correct detection of the R peaks. There is a domino effect between the detection of P and T waves and the detection of R peaks. This is a common challenge in literature published in this area and it is typically not properly addressed [36,37]. This study provides a positive proof-of-concept for detecting P and T waves in arrhythmic ECG beats and brings to light other interesting perspectives that can be investigated. There is a need to investigate P- and T-waves with different morphologies, e.g., biphasic and inverted to determine if the detector performs efficiently. Additionally, different implementations of the event-related moving average methodology will benefit the analysis of ECG signals on portable, wearable, and battery-operated ECG devices.

Even though Brugada’s syndrome investigation was not an explicit goal of our study, it is worth noting that most of the recordings of the MIT-BIH database were acquired in patients treated by drugs such as flecainide, procainamide and ajmaline. These therapies may lead to several ECG alterations, in particular PVCs and QRS prolongation, which also appear in patients affected by this pathology, and are correctly managed by our method, as confirmed by our results. In addition, our model is not affected by ST segment alterations due to Brugada’s syndrome, as this interval is not directly modeled by our algorithm. As a consequence, potential signal deformations due to filtering do not affect ECG wave detection performance.

## 6. Conclusions 

There is a limitation when evaluating arrhythmic P and T wave detection algorithms, due to the lack of datasets with annotated P and T waves, thus making it even more difficult to compare standard detection algorithms. Using 10 arrhythmic ECG signals from the *MIT-BIH Database,* with a total of 21,702 the algorithm was tested and evaluated. Results show promise and are sufficient in demonstrating real-life scenarios of long-recorded arrhythmic ECG signals with different morphologies. Specifically, the algorithm achieved an SE of 98.05% and a +P of 97.11% for P waves, and an SE of 99.86% and a +P of 99.65% for T waves. Moreover, the proposed algorithm performed efficiently and is notably simple when compared to other well-known algorithms in the P and T wave detection field. The simplicity demonstrated in this approach is advantageous, numerically efficient, and allows for the simultaneous detection of the P and T waves. Combined, these advantages motivate future further investigation of this efficient approach.

## Figures and Tables

**Table 1:** **ECG Sinus Rhythm Event Durations.** The proposed algorithm depends on the estimate of the event duration before processing the ECG signal. The window sizes are set with expected event durations. There are three events of the ECG wave: P, QRS and T waves, all measured from a healthy male adult at a heart rate of 60 beats per minute (bpm). Here, *f*_s_ stands for sampling frequency.

Feature	Normal Value	Normal Limit	Normal duration (*f*_s_ = 360 Hz)
P width	110 ms	± 20 ms	33–47 samples
PQ/PR interval	160 ms	± 40 ms	43–72 samples
QRS width	100 ms	± 20 ms	29–43 samples
QTc interval	400 ms	± 40 ms	130–158 samples

**Table 2:** P and T waves detection performance over 10 records from the MIT-BIH Database.

		P Wave Detection Performance					T Wave Detection Performance				
Record	No. of Beats	TP	FP	FN	SE (%)	+P (%)	TP	FP	FN	SE (%)	+P (%)
100	2274	2274	0	0	100.00	100.00	2274	0	0	100.00	100.00
101	1866	1866	0	0	100.00	100.00	1863	3	0	100.00	99.84
102	2187	2021	87	79	96.37	96.02	2187	0	0	100.00	100.00
103	2084	2076	4	4	99.81	99.81	2084	0	0	100.00	100.00
104	2229	2071	82	76	96.58	96.32	2228	1	0	100.00	99.96
105	2602	2557	33	12	99.53	98.72	2579	15	8	99.69	99.42
106	2026	2013	12	1	99.95	99.41	2013	13	0	100.00	99.36
107	2136	2136	0	0	100.00	100.00	2136	0	0	100.00	100.00
108	1765	1363	244	158	90.56	86.13	1710	36	19	98.91	97.95
109	2533	2342	135	56	97.72	94.67	2532	1	0	100.00	99.96
	21702	20719	597	386	98.05	97.11	21,606	69	27	99.86	99.65

Here, TP stands for true positive, FP stands for false positive, FN stands for false negative, SE stands for sensitivity, +P stands for positive predictivity.

**Table 3:** Comparison of several P and T wave algorithms on the MIT-BIH Arrhythmia Database.

Comparison of P Wave Detection Algorithms					Comparison of T Wave Detection Algorithms				
Algorithm	Method	Data Used	Se (%)	+P (%)	Algorithm	Method	Data Used	Se (%)	+P (%)
Proposed algorithm	Blocks of Interest	10 records	98.05	97.11	Proposed algorithm	Blocks of Interest	10 records	99.86	99.65
Arafat et al. [26]	EMD	10,000 beats	N/R	N/R	Arafat et al. [26]	EMD	10,000 beats	N/R	N/R
Diery [27]	Wavelet	39 records (10 s each)	N/R	N/R	Ktata et al. [28]	Wavelet	Selected segments	N/R	N/R
Mahmoodabadi et al. [29]	Wavelet	Selected segments	51.69	53.64	Krimi et al. [30]	Wavelet	Selected beats	94.65	N/R
Ktata et al. [28]	Wavelet	Selected segments	N/R	N/R	Sun et al. [31]	Multiscale morphological derivative	Selected segments	T_ON_ = 99.8 T_OFF_ = 99.6	N/R
Sun et al. [31]	Multiscale derivatives	Selected segments	P_ON_ = 97.2 P_OFF_ = 94.8	N/R	Goutas et al. [32]	Fractional differentiation	Selected segments	N/R	N/R
Goutas et al. [32]	Fractional differentiation differentiation	Selectedsegments	N/R	N/R	Sun et al. [31]	Multi-scale morphological derivative	Selected segments	N/R	N/R

Here, TP stands for true positive, FP stands for false positive, FN stands for false negative, SE stands for sensitivity, +P stands for positive predictivity, EMD stands for empirical mode decomposition, N/R stands for not reported.
